# 早期非小细胞肺癌患者立体定向放射治疗的预后分析

**DOI:** 10.3779/j.issn.1009-3419.2023.102.13

**Published:** 2023-04-20

**Authors:** YU Lu, LI Junyi, GAO Miaomiao, WANG Xiaofeng, BAI Hui, GUAN Yong, YUAN Zhiyong

**Affiliations:** 300060 天津，天津医科大学肿瘤医院，国家恶性肿瘤临床医学研究中心，天津市恶性肿瘤临床医学研究中心; Tianjin Medical University Cancer Institute & Hospital, National Clinical Research Center for Cancer, Tianjin’s Clinical Research Center for Cancer, Tianjin 300060, China

**Keywords:** 肺肿瘤, 立体定向放射治疗, 预后, Lung neoplasms, Stereotactic body radiation therapy, Prognosis

## Abstract

**背景与目的** 随着人口老龄化和肺癌筛查受重视程度的提高，近年来早期肺癌就诊量呈上升趋势。其病理类型以非小细胞肺癌（non-small cell lung cancer, NSCLC）为主，可被划分为可手术早期肺癌和不可手术早期肺癌。立体定向放射治疗（stereotactic body radiation therapy, SBRT）是不可手术早期NSCLC的首选治疗方法。本研究旨在探讨我院早期NSCLC患者行SBRT后的预后及其影响因素，以期提高早期NSCLC患者接受SBRT后的生存期。**方法** 收集2010年8月-2020年8月在我院接受SBRT的早期NSCLC患者的临床资料及随访情况，采用Kaplan-Meier法评估预后，采用Cox比例风险模型进行多因素分析寻找影响预后的因素。**结果** 共纳入165例患者，中位随访时间为43.2（范围：4.8-132.1）个月。1年、2年、5年局部控制（local control, LC）率分别为98.1%、94.8%和86.5%，卡氏功能状态评分（Karnofsky performance status, KPS）>80分是LC的独立预后因素（P=0.02）；1年、2年、5年总生存（overall survival, OS）率分别为97.6%、93.0%和68.9%，生物等效剂量（biological equivalent dose when α/β=10, BED_10_）>132 Gy是OS的独立预后因素（P=0.04）；1年、2年、5年无进展生存（progression-free survival, PFS）率分别为93.3%、79.5%和55.3%；1年、2年、5年无远处转移生存（distance metastasis free survival, DMFS）率分别为94.5%、83.2%和58.4%，BED_10_>150 Gy是DMFS的独立预后因素（P=0.02）；1年、2年、5年区域控制（region control, RC）率分别为98.8%、95.4%和87.9%。**结论** SBRT治疗早期NSCLC疗效好，KPS>80分是影响LC的独立预后因素；BED_10_>132 Gy是OS的独立预后因素；BED_10_>150 Gy是DMFS的独立预后因素。

## Abstract (en)

**Background and objective** With the aging of the population and the increased importance of lung cancer screening, the number of early-stage lung cancer patients has been on the rise in recent years, which can be classified into operable early-stage lung cancer and inoperable early-stage lung cancer. The most common pathological type is non-small cell lung cancer (NSCLC). Stereotactic body radiation therapy (SBRT) is the optimal treatment for inoperable early-stage NSCLC. The aim of this study was to investigate the prognosis of early-stage NSCLC patients treated with SBRT and its influencing factors in order to reduce the side effects of radiotherapy and improve the survival and quality of life. **Methods** Clinical data and follow-up outcomes of early-stage NSCLC patients treated with SBRT in our hospital from August 2010 to August 2020 were collected. Kaplan-Meier method was used to assess the prognosis, and the Cox proportional risk model was used for multivariate prognostic analysis. **Results** A total of 165 patients were included with a median follow-up time of 43.2 (range: 4.8-132.1) mon. The local control (LC) rates at 1-yr, 2-yr and 5-yr were 98.1%, 94.8% and 86.5% respectively. Karnofsky performance status (KPS) score greater than 80 was an independent prognostic factor for LC (P=0.02). The overall survival (OS) rates at 1-yr, 2-yr and 5-yr were 97.6%, 93.0% and 68.9% respectively. A biological equivalent dose when α/β=10 (BED_10_) greater than 132 Gy was an independent prognostic factor for OS (P=0.04). Progression-free survival (PFS) rates at 1-yr, 2-yr and 5-yr were 93.3%, 79.5% and 55.3% respectively. The distance metastasis free survival (DMFS) rates at 1-yr, 2-yr and 5-yr were 94.5%, 83.2% and 58.4% respectively. BED_10_ greater than 150 Gy was an independent prognostic factor for DMFS (P=0.02). The regional control (RC) rates at 1-yr, 2-yr and 5-yr were 98.8%, 95.4% and 87.9% respectively. **Conclusion** SBRT is effective in treating early-stage NSCLC. KPS greater than 80 is an independent prognostic factor for LC; BED_10_ greater than 132 Gy is an independent prognostic factor for OS; BED_10_ greater than 150 Gy is an independent prognostic factor for DMFS.

**【Competing interests】**The authors declare that they have no competing interests.

肺癌是世界上死亡率最高的肿瘤之一，依据病理类型可分为小细胞肺癌和非小细胞肺癌（non-small cell lung cancer, NSCLC），其中NSCLC占80%-85%^[1]^。15%-20%的NSCLC患者初诊时处于早期，即没有区域淋巴结转移和远处转移（distant metastasis, DM）^[2]^。随着人口老龄化的进程和人们对疾病筛查的重视，初诊时处于肺癌疾病早期的患者所占比例逐年升高^[3]^。根据治疗手段不同，可分为可手术的早期肺癌和不可手术的早期肺癌。对于不可手术的早期NSCLC患者，其标准治疗是立体定向放射治疗 （stereotactic body radiation therapy, SBRT）^[4,5]^。SBRT对肿瘤组织进行大剂量低分次照射，周围正常组织受量低^[6,7]^。本研究回顾性分析了在我院行SBRT的早期NSCLC患者的预后及其影响因素。

## 1 材料与方法

### 1.1 资料收集

本研究回顾性收集了2010年8月-2020年8月在我院行SBRT的165例早期NSCLC患者的临床资料。纳入标准（SBRT前）：（1）肿瘤直径≤5 cm，经正电子发射计算机断层显像（positron emission tomography/computed tomography, PET/CT）或胸腹CT、全身发射型计算机断层扫描（emission computed tomography, ECT）等检查未发现有淋巴结转移和DM；（2）穿刺或支气管镜等活检病理为NSCLC；（3）未接受任何抗肿瘤治疗；（4）临床资料完整；（5）生物有效剂量（biologically effective dose when α/β=10, BED_10_）≥100 Gy。排除标准：（1）其他肿瘤病史；（2）肺部病灶>1个；（3）肿瘤直径>5 cm或有淋巴结或DM；（4）既往接受过放疗、化疗等抗肿瘤治疗。收集患者的年龄、性别、吸烟状态、Karnofsky功能状态评分（Karnofsky performance status, KPS）、T分期（根据美国癌症联合会第8版分期标准）、肿瘤直径、肿瘤位置、病理类型、放疗总剂量、分割次数及BED_10_。本研究经过医院伦理委员会批准，患者及家属均知情同意。患者一般资料见表1。中位随访时间为43.2（范围：4.8-132.1）个月。

**表1:** 165例行SBRT的早期NSCLC患者基线资料

Index	Median (range)/n (%)
Age (yr)	71 (43-87)
Gender	
Female	70 (42.4)
Male	95 (57.6)
Smoke	
Yes	103 (62.5)
No	62 (37.5)
KPS	80 (70-100)
T stage	
T_1_	105 (63.6)
T_2_	60 (36.4)
Tumor size (cm)	2.6 (0.8-5.0)
Location	
Peripheral	153 (92.7)
Central	12 (7.3)
Histology	
Adenocarcinoma	108 (65.5)
Squamous cell carcinoma	57 (34.5)
Single fraction radiation dose (Gy)	12 (6-20)
Number of fractions	5 (3-7)
Total dose (Gy)	60 (50-60)
BED_10_ (Gy)	132 (100-180)

NSCLC: non-small cell lung cancer; SBRT: stereotactic body radiation therapy; KPS: Karnofsky performance status; BED_10_: biological equralent dose when α/β=10.

### 1.2 治疗方法

患者采取仰卧定位。对于病灶或病灶周围置入金标的患者，采用射波刀标准体垫定位；未置入金标者根据肿瘤位置及患者全身情况等选用固定方式，病灶位于上中肺的患者通常采用头颈肩网固定，病灶位于下肺的患者通常采用胸网固定。在患者自由呼吸状态屏气采集定位CT（Philips, Brilliance Big Bore）图像，层厚1.25 mm，扫描范围为环状软骨至膈肌下缘。在肺窗上勾画大体肿瘤体积（gross target volume, GTV），选择合适的窗宽窗位勾画心脏、大血管、食管、脊髓、臂丛等危及器官。所有GTV均先由低年资医生进行勾画，再由高年资医生进行逐层修改。对于肺部置入金标的患者，根据X线透视下平静呼吸时金标动度的范围进行外扩形成计划靶区体积（planning target volume, PTV），对于未置入金标患者，定位CT扫描时需要同时扫描4D-CT。将重建最大密度投影图像与定位CT融合后进行GTV勾画，再各向外放3 mm-10 mm形成PTV。照射过程中，133例患者采用呼吸同步追踪技术，32例患者采用X sight追踪技术。照射处方剂量范围：50 Gy-60 Gy；等剂量线范围：64%-83%；分割次数范围：3次-7次。详细的剂量分割模式见表2。治疗系统：美国Accuray公司的第三代射波刀治疗系统（CK-G3）。

**表2:** 165例早期NSCLC患者行射波刀SBRT治疗的剂量分割模式

Radiotherapy dose/ Number of fractions	n (%)	BED_10_ (Gy)
60 Gy/3 F	16 (9.70)	180.0
60 Gy/4 F	29 (17.6)	150.0
60 Gy/5 F	60 (36.4)	132.0
60 Gy/6 F	17 (10.3)	120.0
56 Gy/7 F	11 (6.7)	100.8
55 Gy/5 F	4 (2.4)	115.5
54 Gy/3 F	19(11.5)	151.2
54 Gy/6 F	3 (1.8)	102.6
51 Gy/3 F	3 (1.8)	137.7
50 Gy/5 F	3 (1.8)	100.0

### 1.3 随访及预后评价

随访起始点为SBRT治疗第1天，第1年-2年每2个月-3个月随访一次，第3年-4年每半年随访一次，第5年开始每年随访一次。随访截止至2022年10月1日。总生存期（overall survival, OS）为SBRT治疗第1天开始到死亡或末次随访时间，无病生存期（disease-free survival, DFS）为到第一次疾病进展（progressive disease, PD）的时间。根据实体瘤疗效评价标准1.1（Response Evaluation Criteria in Solid Tumours 1.1, RECIST 1.1）对预后进行评价，局部复发（local recurrence, LR）定义为原发灶所在肺叶复发，区域复发（regional recurrence, RR）定义为纵隔、肺门或锁骨上淋巴结转移，DM为除外LR和RR之外的复发。放射损伤分级按照美国肿瘤放射治疗协作组（Radiation Therapy Oncology Group, RTOG）放射损伤标准评价。

### 1.4 统计学方法

采用MedCalc 19.5.6进行数据分析。生存分析采用Kaplan-Meier法，对数秩检验分析组间差异，Cox比例风险回归模型进行多变量分析。双侧P<0.05为差异有统计学意义。

## 2 结果

### 2.1 失败模式

共60例（36.4%）患者发生PD，中位PD时间为22.1（范围：2.2-82.7）个月；17例（10.3%）患者发生LR，中位局部控制（local control, LC）时间为24.1（范围：5.7-82.7）个月；40例（24.2%）患者发生死亡事件，中位死亡时间为35.9（范围：27.2-42.2）个月；因肺癌死亡18例，其中因DM死亡15例，因LR死亡2例，因RR死亡1例；因其他疾病死亡16例，死亡原因不明6例。54例（32.7%）患者发生DM，中位DM时间为24.5（范围：2.2-82.7）个月；15例（9.1%）患者发生RR，中位RR时间为28.5（范围：5.7-83.6）个月。33例患者仅发生DM，4例患者仅发生LR，2例患者仅发生RR，8例患者同时发生DM和LR，8例患者同时发生DM和RR，5例患者同时发生DM、LR和RR（图1）。

**图 1:**
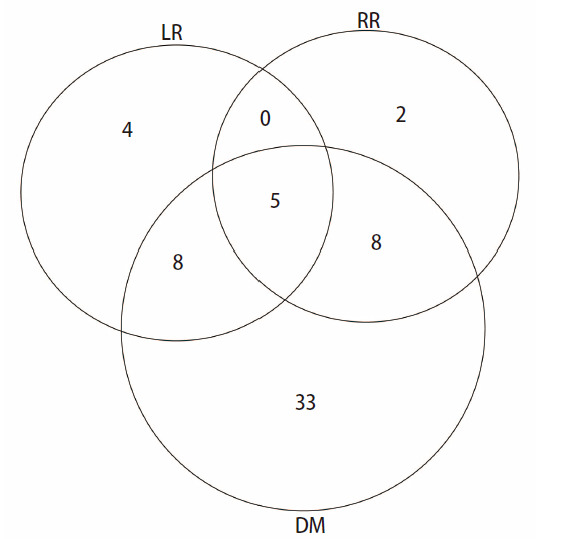
165例早期NSCLC患者行SBRT治疗后各失败模式患者例数

### 2.2 预后及预后因素分析

单因素及多因素分析见表3。全组1年、2年、5年LC率分别为98.1%、94.8%和86.5%，单因素分析显示影响LC的因素为KPS（P<0.01）、肿瘤位置（P<0.01）和T分期（P=0.01），多因素分析发现KPS>80分是影响LC的独立预后因素（P=0.02）。1年、2年、5年OS率分别为97.6%、93.0%和68.9%，中位OS为96.1（95%CI: 84.2-103.3）个月，单因素分析显示影响OS的因素为T分期（P=0.01）和BED_10_（P=0.01），多因素分析发现BED_10_>132 Gy是OS的独立预后因素（P=0.04）。

**表3:** 不同临床结局的单因素及多因素分析

Index	Univariate analysis			Multivariate analysis	
	HR (95%CI)	P		HR (95%CI)	P
LC					
KPS (>80 vs ≤80)	0.22 (0.08-0.57)	<0.01		0.09 (0.01-0.69)	0.02
Tumor size (>2.6 cm vs ≤2.6 cm)	2.36 (0.91-6.12)	0.08		-	-
Location (Central vs Peripheral)	38.81 (4.61-326.74)	<0.01		2.88 (0.86-9.62)	0.09
T stage (T_2_ vs T_1_)	3.78 (1.39-10.30)	0.01		2.82 (0.99-8.05)	0.06
OS					
Age (>71 yr vs ≤71 yr)	1.86 (0.99-3.49)	0.06		1.60 (0.81-3.16)	0.18
Location (Central vs Peripheral)	4.17 (0.89-19.60)	0.07		1.55 (0.51-4.69)	0.44
Histology (SCC vs ADC)	1.76 (0.92-3.38)	0.09		1.56 (0.82-2.95)	0.17
T stage (T_2_ vs T_1_)	2.37 (1.23-4.54)	0.01		1.77 (0.93-3.39)	0.08
BED_10_ (>132 Gy vs ≤132 Gy)	0.40 (0.21-0.75)	0.01		0.45 (0.20-0.99)	0.04
PFS					
T stage (T_2_ vs T_1_)	1.73 (1.00-2.98)	0.04		1.43 (0.85-2.42)	0.18
BED_10_ (>150 Gy vs ≤150 Gy)	0.49 (0.27-0.89)	0.02		0.45 (0.20-1.00)	0.06
DMFS					
Tumor size (>2.6 cm vs ≤2.6 cm)	1.62 (0.95-2.77)	0.08		-	-
T stage (T_2_ vs T_1_)	1.76 (0.99-3.13)	0.06		1.41 (0.81-2.44)	0.22
BED_10_ (>150 Gy vs ≤150 Gy)	0.43 (0.23-0.80)	0.01		0.34 (0.13-0.86)	0.02

SCC: squamous cell carcinoma; ADC: adenocarcinoma; DMFS: distance metastasis free survival.

1年、2年、5年无进展生存（progression-free survival, PFS）率分别为93.3%、79.5%和55.3%，中位PFS为68.2（95%CI: 56.8-82.7）个月（图2），单因素分析显示影响PFS的因素为T分期（P=0.04）和BED_10_（P=0.02），多因素分析未发现明显影响因素。1年、2年、5年DMFS率分别为94.5%、83.2%和58.4%，中位DMFS为69.3（95%CI: 56.8-82.7）个月，单因素分析显示影响DMFS的因素为BED_10_（P=0.01），多因素分析发现BED_10_>150 Gy是DMFS的独立预后因素（P=0.02）；1年、2年、5年区域控制（region control, RC）率分别为98.8%、95.4%和87.9%，单因素及多因素分析未发现影响因素。

**图 2:**
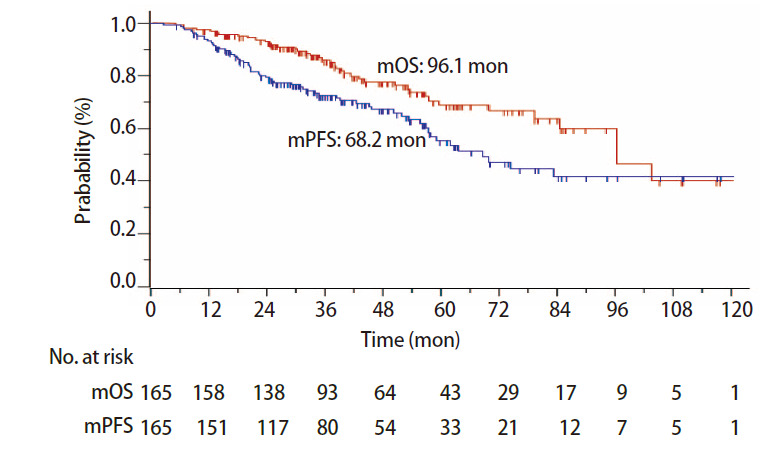
165例行SBRT的早期NSCLC患者的OS及PFS生存曲线

### 2.3 不良反应

所有患者耐受良好，无3级以上的不良反应。7例（4.2%）患者有乏力的表现，5例（3.0%）患者出现1级的放射性肺纤维化，无需治疗。2例（1.2%）患者出现2级的放射性纤维化，经吸氧、化痰等对症治疗后好转。3例（1.8%）患者出现1级放射性肺炎，无需治疗。3例（1.8%）患者出现了2级放射性肺炎，经吸氧镇咳化痰对症治疗后好转。

## 3 讨论

SBRT是不可手术或者拒绝手术的早期NSCLC患者的首选治疗方法，治疗效果好。本中心基于电视胸腔镜手术（video-assisted thoracoscopic surgery, VATS）180例与130例行SBRT的早期NSCLC进行疗效比较，通过倾向评分匹配配对71对，分析显示VATS与SBRT两组OS率、PFS率、DMFS率和RC率均无明显差异，而治疗相关毒性反应SBRT组低于手术组，说明SBRT可取得与手术类似的疗效，且毒性反应更低，具有良好的应用前景^[7]^。SBRT治疗早期肺癌的临床疗效已通过多项前瞻性研究^[8⇓-10]^得到证实，研究中2年LC率为85%-96%。本研究中，2年LC率为94.8%，临床结果与上述前瞻性研究的结果相当。Bae等^[11]^汇总了运用光子和质子治疗早期NSCLC 202例，5年LC率为90.1%，5年PFS率为60.7%，5年OS率为50.8%。而本研究5年LC率为86.5%，5年PFS率为55.3%，5年OS率为68.9%；LC率、PFS率稍差，OS率较好，产生的原因与前者均采用60 Gy/4 F的剂量模式而本研究仅有29例（17.6%）采用60 Gy/4 F的剂量分割模式有关。实施SBRT可采用的放疗技术包括三维适形放疗（three-dimensional conformal radiation therapy, 3D-CRT）、调强放疗（intensity modulated radiation therapy, IMRT）、Cyberknife等。Diamant等^[6]^比较了217例行常规放疗和205例行射波刀的早期NSCLC患者的LC时间和DMFS时间，结果表明射波刀治疗后患者的LC时间和DMFS时间优于常规放疗治疗的患者。射波刀计划中PTV周围剂量下降更慢，对微小残存病灶有更大的致死作用，从而降低DM^[6,12]^。

本研究中最常见的复发模式是DM，32.7%发生DM，中位DM时间24.5个月；DM中位时间均在SBRT后2年-3年内，因此SBRT治疗后前3年的规律复查对于远处转移的监测十分重要。Videtic等^[12]^报道229例患者单次分割SBRT治疗早期NSCLC的长期临床结果，中位随访时间为74.6个月，DM率为16.6%。相对于本研究DM发生率比较低，或与其纳入的患者中32.8%为无病理状态有关，不排除纳入良性结节患者的可能。关于患者的死亡原因，与纳入参数少和样本量小有关。将发生PD、发生DM、发生LR、发生RR纳入单因素分析，发现发生DM（HR=2.24, 95%CI: 1.15-4.35, P=0.02）、发生PD（HR=2.51, 95%CI: 1.31-4.84, P=0.01）是影响患者OS的因素；也与其他疾病致死有关：文中因其他疾病死亡率为9.7%，具体有心脏病、脑梗死、多器官衰竭等；还与患者基础疾病相关：生存分析发现既往无基础疾病组OS优于患基础疾病组（HR=2.65, 95%CI: 1.09-6.45, P=0.03）；Baine^[13]^汇总27,734例行SBRT的早期NSCLC，证明Charlson合并症指数评分3分是OS的独立预后因素，均说明基础疾病影响OS。早期NSCLC行SBRT后主要的失败和死亡原因为DM，因此本机构后续会根据患者的情况来安排更合适的全身治疗。这些复发患者大部分体质比较差，耐受全身化疗的可能性不高，另外患者自身全身化疗的有效率不一定高。所以后续的巩固治疗可能为免疫治疗。目前开展的PACIFIC-4研究为评价Durvalumab与SBRT联合与单用SBRT在早期NSCLC患者的疗效，其研究结果会对复发患者的治疗选择有明示的作用。

既往研究报道性别（女性）、最大标准摄取值（maximum standardized uptake value, SUVmax）>5.5、肿瘤大小和第一秒用力呼吸容积（forced expiratory volume in one second, FEV_1_）是LR的独立预后因子^[14⇓-16]^。本研究中，KPS>80分是影响LC的独立预后因素（P=0.02），该结果可作为影响LC的预后因素的补充。另外，分析显示T分期T_2_（P=0.01）、BED_10_>132 Gy（P=0.01）的患者具有显著更好的OS，而BED_10_>132 Gy是OS的独立预后因素（HR=0.45, 95%CI: 0.20-0.99, P=0.04）。单因素分析发现影响LC和OS的因素不同，是因为T分期、肿瘤直径和BED_10_等因素相互作用、共同影响LC与OS。T分期由肿瘤直径和肿瘤侵犯部位共同决定。回顾性研究^[17]^显示，当肺部病灶肿瘤直径>3 cm时，病灶BED_10_>130 Gy的患者LC更好，从而可能进一步影响OS。在实际临床工作中，当病灶肿瘤直径较大时，本中心医师通常会给予更大的BED_10_，但当肿瘤位置靠近肺门或纵隔内危及器官时，为避免增加患者不良反应发生的风险，此时病灶BED_10_通常不会达到130 Gy。此外，若肿瘤侵犯叶尖裂、胸壁等，DM与RR复发风险可能更高。这可能使得尽管肿瘤直径不显著影响LC，但与肿瘤位置相关的T分期对LC具有显著影响。Klement等^[18]^对1,434例行SBRT的早期NSCLC患者分析发现BED_ave_[BED_min_（PTV周边最小剂量）与BED_max_（1% PTV最大剂量）的平均值]是LR风险的最佳预测因素[曲线下面积（area under the curve, AUC）=0.74]。本研究中BED_10_接近BED_min_，数值低于BED_ave_，这可能是BED_10_对LC无显著影响的原因。支持BED_10_影响LC的文献尚缺，BED_10_与LC的关系是本中心正在探究的课题之一。

本研究BED_10_>132 Gy是OS的独立预后因素（P=0.05）。Moreno等^[19]^发现接受较低BED_10_（100 Gy-129 Gy）的患者与接受较高BED_10_（>130 Gy）的患者相比具有显著较差的OS（P=0.03）。BED_10_对预后的影响程度受肿瘤T分期影响。例如，较低的BED_10_（100 Gy-110 Gy）即可使T_1_分期的患者明显获益，而更高水平的BED_10_（≥130 Gy）对T_2_以上分期的患者尤为重要。基于Moreno等的研究^[19]^，T_2_分期且肿瘤直径>3 cm时，BED_10_≥130 Gy较<130 Gy可使预后情况显著改善。本研究中，T_2_分期患者占36.4%，BED_10_>130 Gy占76.9%，由于BED_10_≤130 Gy的患者较少，以130 Gy为分组阈值时，较高BED_10_组与较低BED_10_组生存无显著差异。当以BED_10_>110 Gy为分界进行生存分析时，结果显示较高BED_10_组与较低BED_10_组存在生存差异（HR=0.16, 95%CI: 0.05-0.54, P=0.01）。总之，对于T_2_以上的患者或者肿瘤直径>3 cm以上的患者，医生采用的BED_10_值会随之变化。对于BED_10_≤132 Gy患者，OS较差，会受益于SBRT后的靶向治疗、免疫治疗等全身治疗。关于早期肺癌SBRT后的辅助治疗，Kann等^[20]^匹配了接受SBRT和含铂双药化疗患者与只接受SBRT的患者，发现辅助化疗是SBRT后RC和DMFS的独立预后因素，Ernani等^[21]^也证实对于肿瘤直径≥4 cm的患者，与单独SBRT相比，SBRT伴辅助化疗与OS的改善相关。这些文章提示辅助化疗有助于SBRT后的患者预后，尤其是高危患者。因此，对于本研究LR风险较高的KPS≤80分患者及OS预后较差的BED_10_≤132 Gy患者，SBRT后的辅助系统治疗是可行的，当然，这需要前瞻性的临床试验来证实。本研究中早期NSCLC行SBRT后不良反应发生率较低，与肺部组织受照射体积剂量限量低有关。Benedict等^[22]^汇总的研究中剂量分割60 Gy/5 F时，病灶所在肺限量标准为V_12.5 Gy_<1,500 cm^3^、V_13.5 Gy_<1,000 cm^3^和V_13.5 Gy_<37%，本研究在此基础上依据患者情况对不同患者个性化地对剂量限值进行优化。此外，较低的不良反应发生率也与治疗过程中根据患者情况决定隔日或隔周末治疗有关；当患者出现轻微咳嗽、咳痰或影像学显示肺部轻微纤维化时，及时予以乙酰半胱氨酸、吡非尼酮或尼达尼布等干预措施可降低严重不良反应的发生率。

本研究也存在一些不足之处。首先，本研究为回顾性研究，在临床资料的获取上不可避免地存在偏倚；其次，本研究纳入的患者样本量及临床因素较少，多因素分析未发现显著影响PFS、RC的临床因素。本研究仅纳入中央型肺癌12例，因此通过1:2倾向性评分匹配进行配对，仅发现肿瘤部位对LC有影响的趋势（P=0.07）。该分析结果与Park等^[23]^纳入111例中央型、140例外周型肺癌的研究结论一致，即肿瘤部位与OS、DFS、RC、DM无关。

综上所述，SBRT治疗早期NSCLC预后好，DM是主要的失败模式；KPS>80分是LC的独立预后因素；BED_10_>132 Gy是OS的独立预后因素；BED_10_>150 Gy是DMFS的独立预后因素。
